# 2-Pyridine Carboxaldehyde for Semi-Automated Soft Spot Identification in Cyclic Peptides

**DOI:** 10.3390/ijms23084269

**Published:** 2022-04-12

**Authors:** Haiying Zhang, Silvi Chacko, Joe R. Cannon

**Affiliations:** Department of Metabolism and Pharmacokinetics, Bristol-Myers Squibb Company, Princeton, NJ 08648, USA; haiying.zhang@bms.com (H.Z.); silvi.chacko@bms.com (S.C.)

**Keywords:** peptide, macrocyclic peptide, proteolysis, proteolytic stability, soft spot, site-specific conjugation

## Abstract

Cyclic peptides are an attractive option as therapeutics due to their ability to disrupt crucial protein–protein interactions and their flexibility in display type screening strategies, but they come with their own bioanalytical challenges in metabolite identification. Initial amide hydrolysis of a cyclic peptide results in a ring opening event in which the sequence is linearized. Unfortunately, the mass of the singly hydrolyzed sequence is the same (M + 18.0106 Da) irrespective of the initial site of hydrolysis, or soft spot. Soft spot identification at this point typically requires time-consuming manual interpretation of the tandem mass spectra, resulting in a substantial bottleneck in the hit to lead process. To overcome this, derivatization using 2-pyridine carboxaldehyde, which shows high selectivity for the alpha amine on the N-terminus, was employed. This strategy results in moderate- to high-efficiency derivatization with a unique mass tag and diagnostic ions that serve to highlight the first amino acid in the newly linearized peptide. The derivatization method and analytical strategy are demonstrated on a whole cell lysate digest, and the soft spot identification strategy is shown with two commercially available cyclic peptides: JB1 and somatostatin. Effective utilization of the automated sample preparation and interpretation of the resulting spectra shown here will serve to reduce the hit-to-lead time for generating promising proteolytically stable peptide candidates.

## 1. Introduction

Although small molecules are still dominant in drug discovery, the modality toolkit available to drug makers is constantly expanding. While small molecules are typically limited to orthosteric binding for direct inhibition, several options are now available that exploit alternative (and allosteric) binding modes. Peptides are one of these options, and typically, the only requirement for hit identification is the ability to abolish a relevant protein–protein interaction (PPI). Recent studies have focused on elucidating massive PPI networks that are replete with data for therapeutic exploitation [1,2,3], especially after superimposing disease-specific mutations and how they perturb normal interaction networks. Using these data, one can see a clear path for identifying hit compounds of interest (HCOI) for progression to lead molecules. Cyclic peptides, specifically, can advance from the HCOI stage very rapidly due to their screening flexibility. Several methods for the generation and screening of large combinatorial peptide libraries have been utilized recently. Heinis and colleagues generated exceptionally large bi-cyclic libraries with high skeletal diversity via an innovative cloning strategy [1]. Derda and co-workers utilized display screening strategies in combination with unnatural pharmacophore insertion into the macrocycle to generate added diversity [2]. Most relevant to this work, Suga and colleagues [3,4,5,6,7,8] pioneered several strategies that utilize a hybrid mRNA-cyclic peptide scaffold that can be used in a display format in conjunction with next-generation sequencing. In this approach, the cyclic peptide is chemically joined to its representative mRNA sequence via a puromycin linker [8]. This structural arrangement enables initial screens to be conducted at the combinatorial library scale (~1 × 10^12^ sequences) since bound peptides will be amplified and subsequently detected via their nucleic acid sequence (as opposed to their non-amplifiable amino acid sequence) [8]. Screening flexibility is an important aspect of any modality, but just like with small molecules, initial screens only identify HCOIs and the path to becoming a viable therapeutic includes optimization of the selectivity, potency, and stability. Unlike small molecules, which are subject to multi-phase oxidative metabolism via predominantly hepatic enzymes, peptides are subject to amide hydrolysis from literally hundreds of (often) ubiquitously expressed proteases and peptidases [9]. Peptide cyclization does enhance stability against amino- and carboxy-peptidases by removing their natural substrates from the intact molecule, but in vitro and in vivo stability studies are still required to fix the metabolic ‘soft spot’, the site of initial amide hydrolysis [10]. For a linear peptide, soft spot identification is straightforward with a known sequence and follows bioinformatic paradigms outlined over several decades in proteomics. The change in mass observed at a very early time point should be attributed to internal hydrolysis, where the mass shift correlates to a subsequence in the intact molecule from either terminus, and this can be identified computationally by conducting a massively error-tolerant search against the parent sequence [11]. The situation becomes much more complicated for the identification of soft spots in cyclic peptides. Hydrolysis of a cyclic peptide, irrespective of its position, results in an M + 18.0106 Da mass shift, and only serves to linearize the peptide. The practical result of the first hydrolysis step in cyclic peptide metabolism is that every possible site of hydrolysis is isomeric with every other possible spot. Amide hydrolysis facilitates targeted MS/MS methods for tracking soft spot formation but interpreting the complex fragmentation spectra is not straightforward, particularly if multiple soft spots exist and co-elute chromatographically. Most tandem spectra are acquired using collision-based fragmentation methods that are known to produce internal fragments, which confound the assignment of true peptide termini after ring opening [12,13]. The complications in interpretation arise from low mass ions that should be indicative of the first amino acids. In cases where internal fragments are formed, the internal ions with low mass can erroneously be identified as terminal residues. This phenomenon can highlight internal sequence tags as terminal tags, resulting in misassignment of the ring opening soft spot. Contrary to approaches used in proteomics on linear peptides with specificity known a priori, in this scenario, the site of enzymatic hydrolysis is unknown, and all but the cleanest spectra make interpretation and analysis difficult. 

To address this problem, we employed site specific labeling at the newly formed N-terminus after initial hydrolysis. This approach utilizes 2-pyridine carboxaldehyde (2PCA) as a derivatization reagent due to its specificity for the α-amine present at the N-terminus, and lack of stable conjugation with ε-amines found on Lys side chains [14]. Francis and co-workers were able to show that 2PCA derivatization is an efficient process with all 20 of the naturally occurring amino acids that were tested under aqueous conditions at physiological pH [15]. Importantly, the imidazolidinone derivative that is made following conjugation requires close proximity between the N-terminal amine and the nitrogen atom from the first amide linkage in the chain, precluding long-lived off target reactivity with Lys side chains. The imidazolidinone formed by 2PCA conjugation imparts a unique residual mass (C_6_H_3_N + 89.0265 Da) that can be used to diagnostically assign low mass ions that are similar to typical b- and y-type ions observed in peptide tandem mass spectra after collisional activation. The utility of this approach was demonstrated on a commercially available cyclic peptide after incubation with a commonly employed matrix for peptide drug stability assays.

## 2. Results and Discussion

### 2.1. Selectivity

**Figure 1:**
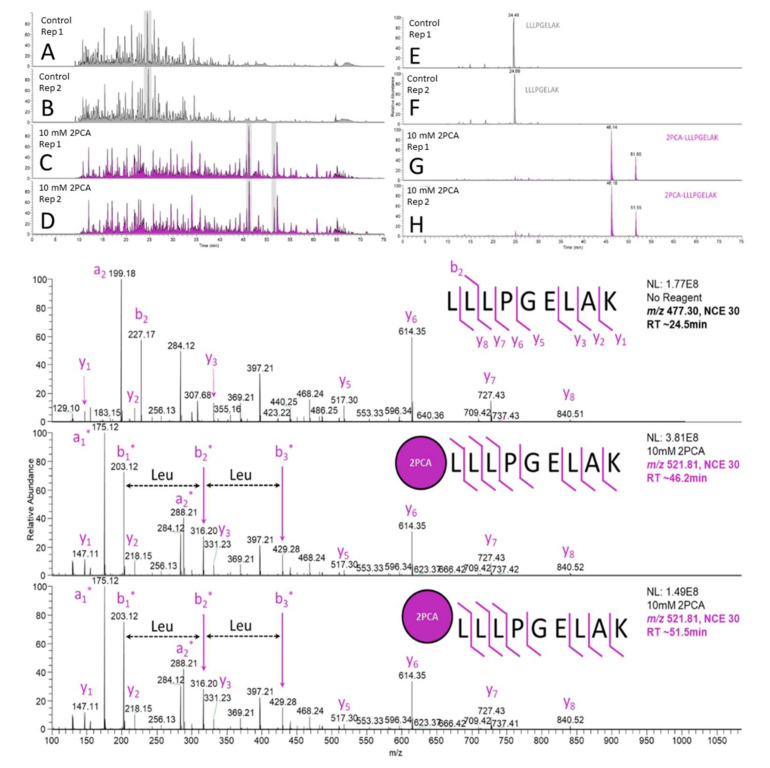
Base peak chromatograms of a HeLa S9 whole cell lysate digest that underwent the entire derivatization procedure in the absence (chromatograms **A** and **B**) and in the presence of the 2PCA reagent (chromatograms **C** and **D**). The extracted ion chromatograms (**E**–**H**) of the most abundant peptides are shown on the right, which showcase their retention time shift and isomerization as a function of 2PCA derivatization. The annotated tandem mass spectra of the most abundant observed peptide and its derivatized versions obtained at both retention times are shown below.

To evaluate the utility of the methods for soft spot identification for cyclic peptides, the reaction was first tested to confirm the selectivity and efficiency on a tryptic digest from HeLa cells. To rapidly generate as many data points as possible to examine the amino acid-specific bias, tryptic peptides were derivatized overnight and analyzed in a high-throughput proteomics experiment. The use of this format allowed rapid testing of thousands of data points with all naturally occurring amino acids (except Pro) situated at the N-terminus. This dataset also enabled a more in-depth look at fragmentation patterns that could be exploited to increase sensitivity and facilitate more automated workflows. Finally, the results were used to construct an analytical method for rapid and sensitive analysis of cyclic peptide soft spots after incubation in a rat luminal contents matrix. From a pairwise comparison (with and without the presence of 2PCA) conducted in the technical duplicate, thousands of peptides and peptide spectral matches were used to discern the derivatization efficiency (Figure 1, Supplementary Tables S1–S7).

Analysis of a derivatized HeLa lysate against a control sample that underwent the same procedure in the absence of 2PCA resulted in confirmation of the high derivatization efficiency reported by the Francis group [15]. On a per N-terminal amino acid basis, only slight bias was observed in the identified peptides, and generally, the derivatization efficiency was moderate to very high across all amino acids tested (Figure S1, Supplementary Table S7). It was also observed that the 2PCA moiety is conjugated in two isomeric forms, resulting in split peaks that are both more strongly retained in an octadecylsilane stationary phase than the linearized unconjugated peptide (Figure 1). The tandem MS spectra of the two peaks were indiscernible from one another, and the major product (the more hydrophilic of the two) was typically more abundant than its isomer (Figure 1, bottom). While peak splitting is not ideal due to a decrease in the limit of detection or quantitation, the sample quantities typically used in in vitro metabolite identification experiments confirm that all but the lowest (relative) abundance metabolites should not be affected by this artefact. Analysis of the product ion spectra of the most abundant peptide allowed confident localization of the +89.0265 Da mass shift to the N-terminus (Figure 1, bottom), with the first 2 amino acids containing both diagnostic fragment ions containing the relevant side chain. Importantly, all y-type ions are shared between the three peptide peaks, indicating N-terminal conjugation.

### 2.2. Diagnostic Fragmentation

**Figure 2:**
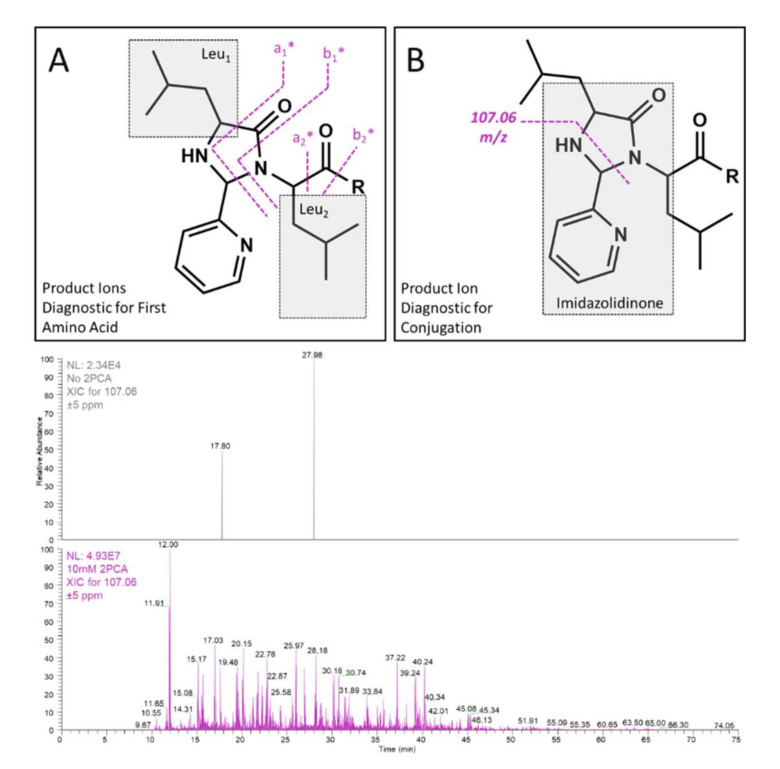
Fragmentation schema that produce diagnostic product ions for the identification of the first amino acid (**A**) and for imidazolidinone formation, in general, are shown (**B**). Extracted ion chromatograms for the *m*/*z* 107.06 ± 5 ppm peak are shown for the control sample in the absence of 2PCA (top chromatogram) and after incubation with 10 mM 2PCA (bottom chromatogram). Note the difference in the base peak abundance.

While it is crucial to feature selective derivatization of the amino acid of interest in these types of studies, it is equally important to ensure that the conjugated product facilitates ease of detection. In this case, the imidazolidinone moiety produced after conjugation provides several crucial product ions that appear at low mass (Figure 2). The primary requirement of the conjugate is to enable simple interpretation of the N-terminal amino acid, and this is facilitated by quadrupole fragmentation to break two distinct bonds across the imidazolidinone (Figure 2A). The two fragments that are observed break the amide backbone on either side of the carbonyl moiety, and result in a diagnostic doublet of 27.9949 Da, analogous to an a- and b-type doublet that is sometimes observed in quadrupole-based fragmentation. Importantly, both of these fragments contain the side chain of the first amino acid and convey the diagnostic residual mass. The additional mass imparted by 2PCA conjugation is 89.0265 Da, and this mass is not shared by any of the 20 most abundant naturally occurring amino acids. The distinction between 2PCA and any natural amino acid is critical to prevent misassignment of 2PCA conjugated fragments as unmodified sequence ions. The third low mass ion generated by imidazolidinone is not specific for the first amino acid but rather for the conjugated product itself (Figure 2B). The fragment, C_6_H_7_N_2_ (*m*/*z* 107.0609), is not an abundant product ion and typically is less than 5–10% of the base peak in any given tandem spectrum. Despite the low abundance, as shown in Figure 2, this fragment imparts orthogonality and is not detected in samples that are not conjugated with 2PCA. Notably, the orthogonality of this product ion enables a more versatile approach to metabolite identification. 

**Figure 3:**
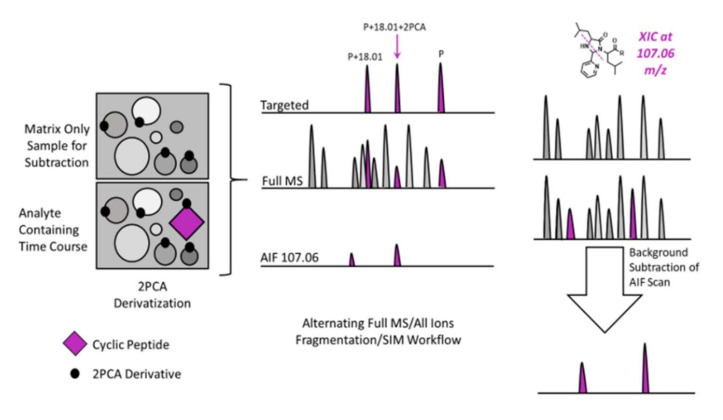
A cartoon representation of the workflow utilized for semi-automated SSID of a commercially available cyclic peptide, JB1. Analyte containing samples and matrix only controls were subjected to MS analysis with unscheduled PRM analysis (for the parent, hydrolyzed parent, and hydrolyzed parent modified by 2PCA), full MS with DDA, and AIF for unbiased observation of the diagnostic imidazolidinone peak. Background subtraction was then performed on the AIF scans to identify analyte-specific 2PCA conjugates.

This allows for easy method development since the first hydrolysis event always changes the mass by 18.01 Da, and the addition of a single 2PCA moiety on the N-terminus changes the mass by an additional 89.0265 Da. Known masses and calculated *m*/*z* values support targeted approaches to confirm that the expected ions are observed and fragmented. The diagnostic peak at 107.0609 Da adds further selectivity and facilitates the observation of 2PCA conjugated metabolites that have undergone more than 1 hydrolysis event. This observation led us to use a multi scan approach for the identification of cyclic peptide metabolites. Specifically, the combination of all ion fragmentation (AIF), in which all ions entering the source across the entire interrogated *m*/*z* range are fragmented simultaneously, a typical MS1 full scan, and targeted analysis of the particular *m*/*z* values of interest (Parent + H_2_O, and Parent + H_2_O + 2PCA) facilitates sequencing of the soft spot(s) and any additional (secondary) metabolites that still contain the 2PCA-modified terminus (Figure 3).

### 2.3. Background Subtraction and AIF Workflow

This three-pronged approach was used to analyze two commercially available cyclic peptides, JB1 and somatostatin, in a typical soft spot identification workflow with automated sample preparation. Both JB1, a 12-mer cyclic peptide with the sequence CYAAPLKPAKSC, and somatostatin, a 14-mer cyclic peptide with the sequence AGCKNFFWKTFTSC (disulfide bound between Cys residues), were subjected to incubation in dilute rat intestinal luminal contents matrix on a liquid handling system. To facilitate unsupervised sample preparation, the enzymatic activity of the matrix was quenched with 1 volume of acetonitrile containing 20 mM 2PCA and incubated overnight. The following day, the samples were injected into the MS for analysis directly with the approach outlined in Figure 3. To increase the throughput and proportion of ‘hands-off’ time, the derivatization step was not quenched, and excess reagent or Schiff bases formed during the incubation were not scavenged by hydrazide (as was performed during the selectivity experiments on the tryptic HeLa lysate). While omitting the hydrazide scavenging step could introduce a decrease in sensitivity due to Schiff base formation on basic sites, the concentrations typically used in in vitro incubations ensure that minor decreases in sensitivity will not have deleterious effects on the workflow as a whole. In this case, the liquid handler utilized was unable to manipulate magnetic beads, but future experiments will incorporate a magnetic-bead-compatible liquid handler to incorporate the scavenging step. The method described has the potential to create more than one 2PCA adduct on a given peptide with basic sites, but the utilization of a targeted approach for the singly modified peptide as outlined here circumvents that complication and ensures MS/MS sampling of the peptide of interest.

**Figure 4:**
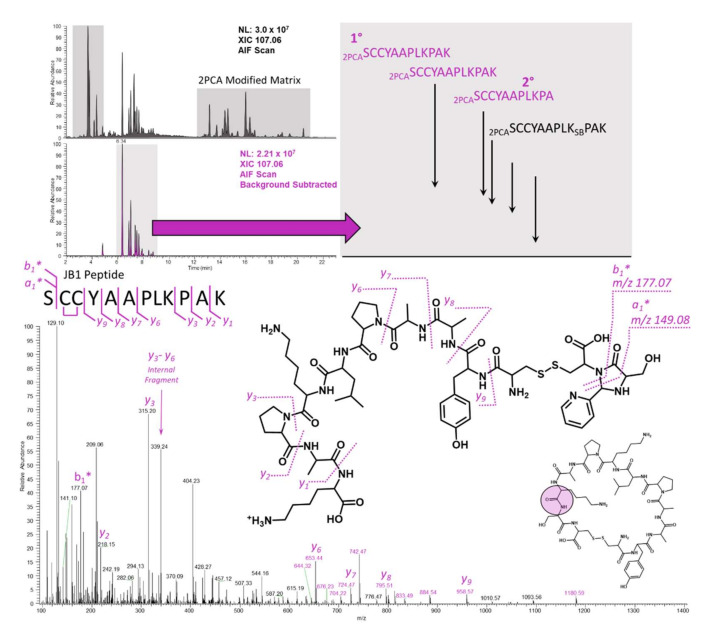
XICs for the unprocessed (top left chromatogram) and background subtracted (bottom left chromatogram) 10 min time points after JB1 incubation in dilute rat intestinal luminal contents. A zoomed-in view (top right) of the background subtracted XIC facilitates simple assignment based on the precursor mass alone. Targeted analysis of the hydrolyzed and 2PCA-modified parent mass results in the easily interpretable spectrum shown on the bottom.

Use of the approach outlined in Figure 3 enabled rapid and simple interpretation of the soft spot for the JB1 and somatostatin peptides. As mentioned earlier, targeted methods for cyclic peptides in soft spot identification can benefit immensely from a priori knowledge of the *m*/*z* values of interest. Surveying the parent, primary hydrolysis product, and 2PCA conjugate masses over the time course facilitates an understanding of the proteolytic picture for the cyclic peptide. Principally, one can confirm that parent reduction over time correlates with an increase in the singly hydrolyzed product(s), and one can confirm that the derivatization efficiency is sufficient for tandem MS sequencing. Simultaneously, monitoring the formation of the 107.0609 *m*/*z* imidazolidinone-specific product ion in an AIF format allows the user to overlay the targeted method with the 2PCA method, and confirm that the 2PCA modification is, in fact, on the analyte and not on a matrix peptide. The utility of the AIF scan with background subtraction is demonstrated in Figure 4. Background subtraction has been used extensively for metabolite identification experiments [16,17,18,19,20], and even in the context of AIF [17]. This precedent method enables rapid discrimination between 2PCA conjugates that are present on matrix molecules and those that are present on the actual cyclic peptide analyte of interest. The selectivity of 2PCA is for α-amines, which are present on the N-terminus of all non-acetylated intact proteins and all internally formed peptides (that have not undergone N-terminal side cyclization). For intact proteins, proteomic studies in yeast and humans have observed that a very high proportion of proteins are acetylated (57% in yeast and 84% in humans) [21], and this is thought to happen co-translationally on the nascent polypeptide chain [22]. Peptides that are formed via proteolysis of matrix proteins by matrix proteases are a much greater concern. This becomes especially important with the harsh matrices that are used for achieving maximal stability. Figure 4 shows the utility of background subtraction for the JB1 peptide analysis by separating out the true 2PCA modified analyte peaks (Figure 4, bottom XIC) from those peptides that are generated from the matrix alone (Figure 4, top XIC). Zooming in on the background subtracted XIC for the diagnostic ion shows several peaks that represent not only the cyclic peptide soft spot but also secondary metabolites that have undergone a subsequent hydrolysis step, in this case clipping of the C-terminal Lysine residue.

### 2.4. JB1

In addition to the simple assignment of the most N-terminal residue, the JB1 peptide serves to illustrate difficulties associated with collision-based fragmentation methods. Prolines are well known in proteomics and mass spectrometry to be favorable sites for fragmentation due to the torsional strain of the cyclic imine [23,24,25]. Internal fragmentation is not uncommon in mass spectrometry and has recently been exploited in top-down protein characterization [26,27]. In the JB1 peptide, collisional activation results in the formation of an internal fragment between the 2 prolines at *m*/*z* 339.24 (Figure 4, bottom). This fragment is composed of the three residues PLK, and is isobaric with an additional internal tripeptide fragment, LKP, also found in the JB1 sequence. In top-down (and even bottom-up) workflows, internal fragments are largely innocuous because the true termini of the protein (or peptide) are known based on the translational stop (or enzymatic specificity for bottom up). In this case, with an unknown site of hydrolysis, internal fragments confound accurate interpretation by suggesting a false start or stop site in the peptide. The diagnostic mass of the 2PCA moiety on the first residue enables simple and rapid interpretation to show that the first residue is indeed a serine. Interestingly, and in the case of the JB1 peptide, an integral mass shift correlating to the mass of a single hydrogen atom was observed. This results in a ~1.0078 Da difference from the calculated mass of 178.0742 Da (observed at 177.0671 Da). Discrepancies like this resulting from hydrogen transfer are not uncommon, particularly in fragmentation mechanisms that are not completely understood [28,29]. Gas phase fragmentation of the 2PCA moiety on a peptide terminus is not completely understood, and comprehension is complicated further by the fact that two bonds are broken across the newly formed imidazolidinone. Alternative mechanisms of gas phase fragmentation (electron based) will be explored in the future. Despite the single hydrogen discrepancy, the resulting spectrum can be confidently interpreted based on a combination of the nearly complete y-ion series (Figure 4, bottom) and the two diagnostic masses associated with the terminal serine. It is important to consider that fragmentation is different from hydrolysis, and the latter imparts substitution of a hydroxyl group on the newly formed C-terminus that changes the mass of the entire series of the resulting x-, y-, or z-type fragments.

### 2.5. Somatostatin

**Figure 5:**
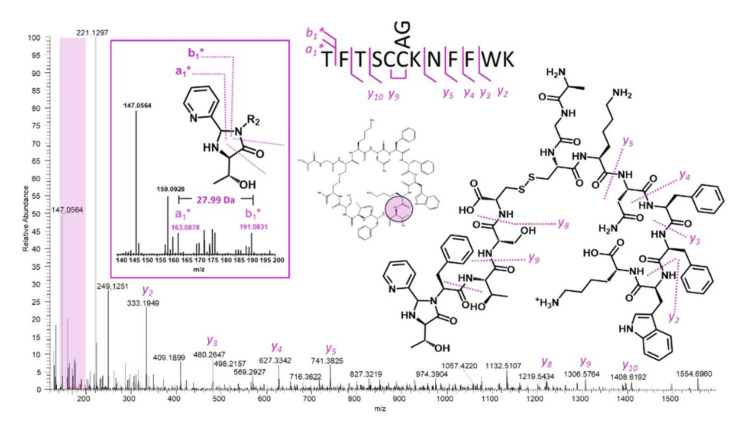
MS/MS analysis of the hydrolyzed and 2PCA-modified somatostatin parent mass results in the easily interpretable spectrum shown above. The inset shows the two diagnostic ions across imidazolidinone that result in confident identification of the most N-terminal threonine.

Figure 5 illustrates the same approach applied to somatostatin. Careful examination of the low mass region shows that the diagnostic doublet of the pseudo a1- and b1-type ions is present at 191.08 and 163.08 *m*/*z* (Figure 5, inset). This doublet suggests that the most N-terminal residue is threonine. Examination of the somatostatin sequence shows that there are two threonine residues separated by a phenylalanine. While the b-type ion sequence is uninformative in this example, the y-ion series is nearly complete due to sequestered charge at the lysine ε-amine. A very quick comparison of matching C-terminal peaks for the first threonine (followed by phenylalanine) against matches for the second threonine (followed by serine) reveals that only the first one results in a matched sequence. Somatostatin represents an important example because many of the HCOIs generated from mRNA display screens are rich in aromatic residues, and frequently contain the same residues in several positions. As an example, Fushman and Suga utilized an mRNA display to identify linkage-specific ubiquitin binders and identified a 15-mer peptide with high affinity for Lys48 di-ubiquitin, which contained 7 aromatic amino acids (5 tyrosine and 2 tryptophan residues) [30]. While this could be thought of as an extreme example, it is quite common for mRNA display HCOIs to be enriched in aromatic residues, and it is rare for a macrocyclic peptide to not contain any duplicate amino acids. Even with this caveat, the 2PCA approach enables a rapid testable hypothesis for the N-terminal residue, and this, in turn, allows the practitioner to confirm or disprove the hypothesis via C-terminal ion sequencing. 

## 3. Materials and Methods

### 3.1. Materials

HeLa S9 lysate tryptic digest was purchased from ThermoFisher (San Jose, CA, USA). JB1 trifluoroacetate salt (J3705) was purchased from Sigma-Aldrich (St. Louis, MO, USA). Somatostatin-14 peptide (AS-24277) was purchased from Anaspec (Fremont, CA, USA). Rat intestinal luminal contents (through custom order with Sekisui Xenotech (Kansas City, KS, USA)) were obtained from 12 fasted male SD rats, with small intestine tissue procured and contents flushed from the lumen of the duodenum and jejunum.

### 3.2. Complex Mixture Derivatization

In total, 40 μg of HeLa S9 lysate digests were diluted to a concentration of 0.4 mg/mL in 25/75 (*v*/*v*) acetonitrile/20 mM EPPS and split into 3 equal aliquots. To each 30 µL aliquot, 3 µL of either buffer or 2-pyridine carboxaldehyde (2PCA) were added to obtain a final concentration of 10 mM. The digests were incubated overnight at room temperature. The following day, hydrazide-functionalized magnetic beads were washed 3 times in 25/75 (*v*/*v*) acetonitrile/20 mM EPPS buffer and then added to the digest incubations to quench the derivatization and remove unreacted 2PCA after Schiff Base reversion. Derivatized peptides were incubated with magnetic beads for 30 min at 37 °C and the beads were magnetically removed from the supernatants. The supernatants containing the derivatized peptides were evaporated to dryness and resuspended in 0.1% formic acid in water (*v*/*v*).

### 3.3. Metabolite Identification Time Course Automated Sample Preparation

The flushed intestinal luminal contents from fasted rats were pooled and the concentration of protein was determined. The incubation for cyclic peptide metabolism was conducted at 37 °C with a final concentration of 0.02 mg/mL total protein (in 5 mM pH 7.4 phosphate buffer) and with 10 µM of cyclic peptides of interest. The incubation was conducted on a Tecan Freedom EVO liquid handler (Tecan, Morrisville, NC) for automated sample preparation. Aliquots of the incubation were taken at 0-, 2.5-, 10-, and 20-min intervals and were quenched with an equal volume of acetonitrile, which also contained 20 mM 2PCA (10 mM final concentration). The quenched incubation aliquots were centrifuged and were set at room temperature overnight for the 2PCA derivatization to complete.

### 3.4. LC-MS/MS

HPLC was performed using an Acquity UPLC CSH C18 column (1.7 μm particle size, 2.1 mm × 150 mm, Waters, Milford, MA, USA) with a flow rate of 400 μL/min. The HPLC solvents were water and acetonitrile, both containing 0.1% formic acid. The HPLC gradient was initiated at 2% acetonitrile, held for 2 min, and then linearly ramped to 80% acetonitrile in 21 min. After raising the gradient to 98% acetonitrile and maintaining it for 4 min, the gradient was returned to the initial conditions and re-equilibrated for 3 min. The sample injection volume was 20 μL. A Q Exactive Orbitrap mass spectrometer (Thermo Scientific, San Jose, CA, USA) equipped with an electrospray interface was used in-line with the HPLC system described above. The LC/MS data were acquired in positive mode using 2 alternating scanning functions: the first was full MS coupled with data-dependent MS/MS at a normalized collision energy of 30% with an inclusion list targeting the [parent + 18.01 Da] and the [parent + 18.01 Da + 2PCA] metabolite ions; the other was all ion fragmentation (AIF) data obtained at a normalized collision energy of 35%. All data were acquired in centroid format, with full MS in the range of *m*/*z* 400 to 2000 and AIF in the range of *m*/*z* 100 to 1500. The mass resolution was set to 35,000 for full MS and AIF, and 17,500 for data-dependent MS/MS.

### 3.5. Background Subtraction

For background subtraction preparation, the acquired LC/MS data were first processed with a Slicer utility in RecalOffline (Thermo Scientific, San Jose, CA, USA) to extract the AIF data into separate data files. The AIF data files were converted to NetCDF format using the file conversion utilities provided by the instrument vendors. Background subtraction was conducted on the NetCDF format of the AIF datasets. In this proof-of-principle study, where incubation samples were generated with multiple cyclic peptides, the data of samples from other cyclic peptides were used as controls (referred to as “control scans” below) to provide matrix component coverage for the analysis of data of the sample of a given cyclic peptide (referred to as “analyte scans” below). The background subtraction algorithm has been described elsewhere [16,17,18,19]. In brief, the software defined a range of control scans based on a specified time window around an analyte scan (±0.2 min in this study) so that the accurate mass data contained within that window could be considered for matrix ion checking. The dynamic “range of control scans” algorithm was looped throughout the analyte scans in an LC/MS data set. Once the control scans were defined for an analyte scan, the actual subtraction of any ion in the spectrum of the analyte scan was performed by first identifying the same ion in the spectra of the control scans (masses in analyte vs. control data were matched if they fell within a specified mass tolerance window around the analyte masses, which was set to ±10 ppm in this study). The highest intensity of the identified ion in the spectra of the control scans was then determined and multiplied with a specified scaling factor (2) and was subtracted from that of the ion in the spectrum of the analyte scan. After data processing, the output NetCDF file was converted back to the native XCalibur data format for viewing.

## 4. Conclusions

A novel strategy for cyclic peptide soft spot identification that relies on selective derivatization of the newly formed N-terminus after ring opening hydrolysis was presented. The 2PCA method, first proposed by Francis and co-workers for α-amine derivatization under native conditions, was repurposed for in vitro cyclic peptide metabolite identification. By simply adding the 2PCA reagent into our time course quench solution and incubating it overnight, the new strategy fits nearly seamlessly into the automated sample preparation workflow already in place in typical early-stage drug discovery environments. Utilization of the approaches demonstrated herein can potentially decrease the time required for HCOIs to become (in vitro) proteolytically stable candidates from months to weeks. Here, the method was showcased using a tryptic digest of a HeLa whole cell lysate, and then subsequently demonstrated in a real semi-automated time course with two commercially available cyclic peptides, JB1 and somatostatin. Combining the simple N-terminal derivatization with previously optimized background subtraction methods facilitates simple and rapid interpretation for high-confidence soft spot identification. Future work will focus on extending the 2PCA methodology to assay proteolytic stability in vivo.

## Data Availability

Not applicable.

## Supplementary Materials

The following supporting information can be downloaded at: https://www.mdpi.com/article/10.3390/ijms23084269/s1.
