# Supplementary material for: 2-Pyridine Carboxaldehyde for Semi-Automated Soft Spot Identification in Cyclic Peptides
Source: Int J Mol Sci. 2022 Apr 12;23(8):4269. doi: 10.3390/ijms23084269 (PMC9028278; doi:10.3390/ijms23084269)
Supplement: Supplementary file 1 [file ijms-23-04269-s001.zip › ijms-1653958-supplementary.pdf]

2-Pyridine Carboxaldehyde for Semi-Automated Soft Spot Identification in Cyclic Peptides

Haiying Zhang, Silvi Chacko, and Joe R. Cannon

Department of Metabolism and Pharmacokinetics, Bristol-Myers Squibb Company, Princeton, NJ

*Correspondence: joe.cannon@bms.com

Supplementary Information

Supplementary Figure S1. Histogram depicting empirically defined N-terminal amino acid dependence on derivatization efficiency for the 2PCA moiety.
Supplementary Table S1. All SEQUEST identified peptides from HeLa lysate tryptic digest in the absence of 2PCA (1st replicate).
Supplementary Table S2. All SEQUEST identified peptides from HeLa lysate tryptic digest in the absence of 2PCA (2nd replicate).
Supplementary Table S3. All SEQUEST identified peptides from HeLa lysate tryptic digest in the presence of 2PCA (1st replicate).
Supplementary Table S4. All SEQUEST identified peptides from HeLa lysate tryptic digest in the presence of 2PCA (2nd replicate).
Supplementary Table S5. All 2PCA modified SEQUEST identified peptides from HeLa lysate tryptic digest in the presence of 2PCA (1st replicate).
Supplementary Table S6. All 2PCA modified SEQUEST identified peptides from HeLa lysate tryptic digest in the presence of 2PCA (2nd replicate).
Supplementary Table S7. Calculated derivatization efficiency of 2PCA on a per N-terminal amino acid basis.

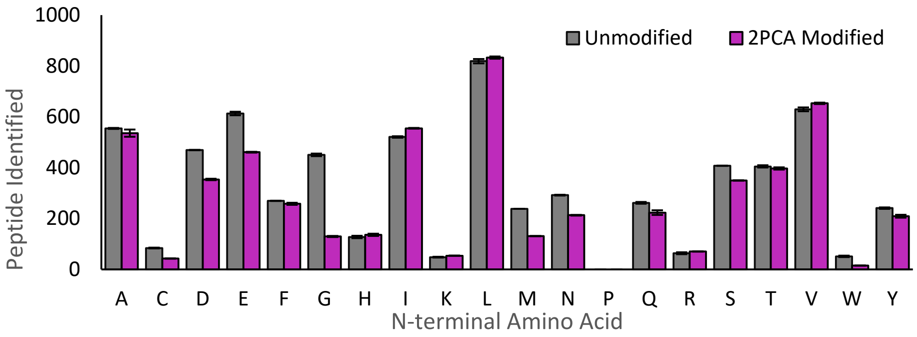

Supplementary Figure S1. Histogram depicting empirically defined N-terminal amino acid dependence on derivatization efficiency for the 2PCA moiety.
